# Molecular signatures of electrochemically reduced peatland dissolved organic matter resolved by liquid chromatography Fourier transform ion cyclotron resonance mass spectrometry

**DOI:** 10.1007/s00216-026-06668-y

**Published:** 2026-07-17

**Authors:** Rania Mobarak, Carsten Simon, Patrick Guth, Klaus-Holger Knorr, Maximilian P. Lau, Oliver J. Lechtenfeld

**Affiliations:** 1 Research Group BioGeoOmics, Department of Environmental Analytical Chemistry, Helmholtz Centre for Environmental Research-UFZ, 04318 Leipzig, Germany; 2 Institute of Landscape Ecology, Ecohydrology & Biogeochemistry Group, University of Münster, 48149 Münster, Germany; 3 Interdisciplinary Environmental Research Centre, Technische Universität Bergakademie Freiberg, 09599 Freiberg, Germany

**Keywords:** Dissolved organic matter (DOM), Direct electrochemical reduction (DER), Liquid chromatography Fourier transform ion cyclotron resonance mass spectrometry (LC-FT-ICR MS), Electron-accepting capacity (EAC), Redox active molecular formulas

## Abstract

Dissolved organic matter (DOM) plays a central role in peatland carbon cycling, where anoxic conditions, redox processes, and thermodynamic factors impact carbon preservation. DOM mediates electron transfer reactions through quinone-like and other redox-active moieties, substantially influencing electron and carbon balances. However, the molecular basis of its electron-accepting and electron-donating capacities remains poorly resolved. Here, DOM from three peatlands was subjected to mild, direct electrochemical reduction (DER; − 0.59 V vs. Ag/AgCl) and analyzed by liquid chromatography coupled to Fourier transform ion cyclotron resonance mass spectrometry (LC-FT-ICR MS). Dissolved organic carbon concentrations measured before and after DER indicated only marginal loss of carbon during reduction. In contrast, electrochemical reduction induced pronounced decreases in FT-ICR MS-derived total ion counts across peat samples, reaching up to 30%, relative to unreduced DOM, while the overall molecular formula space was largely conserved. Changes in signal intensity were negatively associated with double bond equivalents (DBE), indicating that more unsaturated molecular formulas were preferentially affected by DER. In contrast, DBE–O (DBE minus number of oxygen) and modified aromaticity index showed weaker relationships with intensity changes, suggesting that the DBE dependence reflects a broader unsaturation-related response rather than direct reduction of specific functional groups. Across peat DOM, we found average changes in DBE between −0.23 and −0.62 that related to bulk electron-accepting capacity (1000 to 1600 µmol e⁻ (gC)⁻^1^). We identified molecular unsaturation rather than polarity or oxygen content as an important descriptor of DOM redox activity and demonstrate that direct coupling of LC-FT-ICR MS to batch electrolysis can resolve molecular patterns associated with bulk redox behavior.

**Graphical abstract:**

**Figure:**
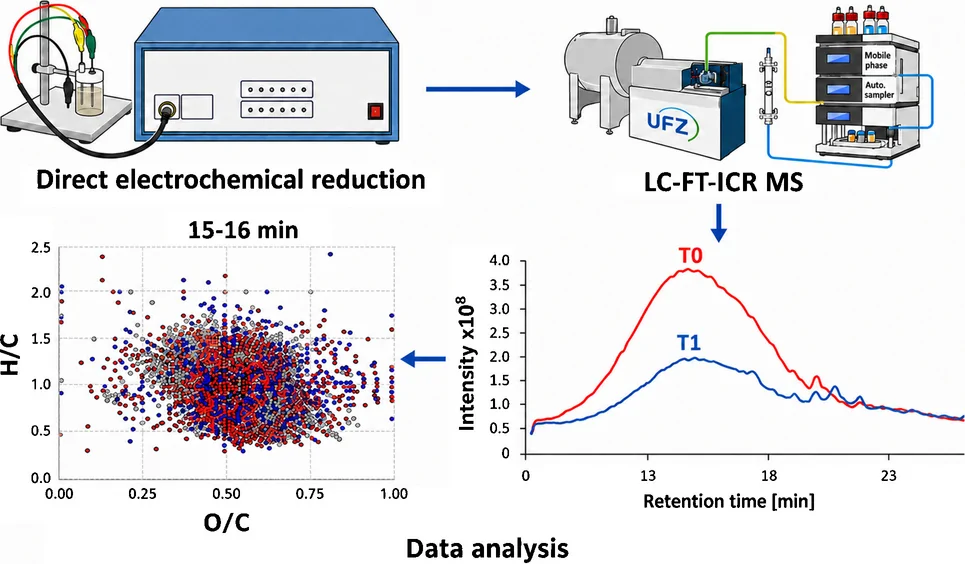

**Supplementary Information:**

The online version contains supplementary material available at https://doi.org/10.1007/s00216-026-06668-y.

## Introduction

Peatlands occupy only around 3–4% of Earth’s land surface, yet they hold a disproportionately large share, nearly one third, of the world’s soil carbon stock, vastly exceeding the carbon stored in land vegetation [1]. Their waterlogged state with persistent anoxic conditions substantially slows down organic matter decomposition, enabling long-term carbon accumulation and stabilization [2, 3]. Within these ecosystems, dissolved organic matter (DOM) constitutes a mobile and sensitive pool of organic carbon. It is the fraction most available for microbial utilization, can readily be exported to aqueous environments, and hence strongly influences carbon cycling and associated climate dynamics. Understanding the biogeochemistry of DOM in peatlands is essential to constrain its role in carbon cycling.

DOM in peat porewater is relatively aromatic and composed of higher molecular weight compounds, reflecting a complex structure and variable lability within the peat matrix [4]. However, molecular-level understanding of how anoxia influences the composition and reactivity of peatland DOM remains limited due to challenges in accurately isolating and characterizing its complex and heterogeneous components [5]. Conversely, specific functional groups and molecular classes determine its biogeochemical role and potential as a terminal electron acceptor in anoxic environments [6]. In particular, DOM in such systems contains redox-active moieties, notably quinones and phenolic compounds, which enable reversible electron transfer processes [7]. These redox reactions are central to peatland biogeochemistry; they regulate microbial respiration, shape pathways of carbon mineralization, control CO₂ emissions, and suppress methane formation, thus collectively mediating key biotic and abiotic interactions in peatlands [8–10].

To understand how DOM mediates electron transfer and contributes to biogeochemical cycling, it is essential to identify and quantify its redox-active functional groups and corresponding electron-donating or electron-accepting capacities. Qualitatively, wet-chemical reduction techniques, such as sodium borohydride (NaBH₄) treatment, which introduces hydride (H^−^) to carbonyl and quinone moieties and converts them predominantly to the corresponding alcohols and hydroquinones, have provided valuable insights into the presence and reactivity of these functional groups in humic substances and DOM [11, 12]. In addition, catalytic hydrogenation approaches, using either homogeneous transition-metal complexes or heterogeneous metal-on-carbon catalysts, have been employed to hydrogenate unsaturated and aromatic structures in lignin- and humic-like matrices, offering complementary constraints on redox-active functional groups under strongly reducing conditions [13–15]. However, such methods are typically conducted under non-environmental conditions and offer limited control over electron-transfer kinetics and quantitative electron balances [16, 17].

Electrochemical (EC) methods provide a quantitative means to probe DOM’s redox properties under controlled conditions, linking molecular structure to reactivity [7, 18]. Direct EC studies performed in aprotic solvents such as dimethyl sulfoxide (DMSO) were instrumental in identifying redox-active moieties and demonstrating direct electron transfer at the electrode interface [19]. Although these systems enable mechanistic insights, they differ substantially from natural aqueous conditions. Direct electrochemical reduction (DER) approaches in aqueous media have also been explored without mediators; however, electron-transfer kinetics are often slow and may limit quantitative resolution. In contrast, mediated electrochemical techniques enable solution-based electron transfer under environmentally relevant conditions [7, 20–22]; however, these approaches provide primarily bulk redox information without molecular-level resolution.

Coupling electrochemical techniques with ultrahigh-resolution Fourier transform ion cyclotron resonance mass spectrometry (FT-ICR MS) enables direct molecular-level resolution of redox transformations within complex DOM mixtures. Changes in individual molecular formulas can be tracked following controlled reduction or oxidation, allowing identification of redox-active compounds beyond bulk-level correlations [20, 21, 23–26]. Such integrative approaches have greatly advanced our molecular-level understanding of DOM composition and its role in environmental redox processes. Although recent work has indirectly connected electrochemical signals with compositional changes in DOM, coupling between electrochemical reduction and FT-ICR MS has not yet been realized. Consequently, linking electrochemically derived redox characteristics to specific molecular transformations within DOM remains a significant analytical challenge [27]. For instance, solid-phase extraction (SPE) is typically needed to conduct FT-ICR MS analysis but can selectively exclude polar and highly functionalized compounds, leading to incomplete molecular representation of extracts measured with FT-ICR MS [28] and bulk EC measurements performed on native DOM. Liquid chromatography (LC) hyphenated with FT-ICR MS provides an alternative approach that maintains the compositional diversity of DOM through direct injection of native samples even at high salt or buffer content [29, 30]. LC-FT-ICR MS further benefits from isomer separation and minimizes matrix effects, overcoming limitations of direct infusion, such as ion suppression, and therefore improves overall sensitivity [29, 31]. Coupling LC-FT-ICR MS with EC methods enables coordinated analysis of electrochemical responses and molecular compositional changes within a unified experimental workflow, improving comparability between redox signals and molecular-level transformations. While molecular detection remains limited to compounds ionizable in electrospray ionization (ESI) in negative mode, this approach effectively captures the dominant acidic and oxygen-rich fraction of peatland DOM, which constitutes the primary redox-active pool under environmental conditions.

To address existing analytical gaps in linking DOM redox characterization via EC methods with molecular-level compositional analysis, this study evaluates the potential of a DER approach coupled with LC-FT-ICR MS. DOM from peatlands was selected because it represents a chemically complex and highly redox-active pool of natural organic matter. Although peatlands are predominantly anoxic and the resident DOM is partially reduced, previous studies have shown that it still contains abundant redox-active functional groups, particularly quinones, phenolics, and semiquinones that participate in reversible electron transfer reactions [15, 32, 33]. These properties make peatland DOM an ideal test material for assessing the capability of electrochemical methods to quantify and induce redox transformations within structurally diverse organic mixtures. We hypothesize that (1) electrochemical reduction induces quantifiable changes in LC-FT-ICR MS signal response of DOM, as reflected by shifts in molecular peak intensities, and (2) these changes are molecularly selective rather than uniformly distributed across DOM. Molecular unsaturation is expected to contribute to the electrochemical response, while oxygen-containing and other redox-active molecular features further shape molecular formula-level intensity changes. Double bond equivalents (DBE), DBE minus number of oxygen (DBE-O), and the modified aromaticity index (AI_mod_) are therefore used as complementary descriptors to evaluate redox-responsive molecular patterns without implying stoichiometric reduction of specific double bonds or functional groups. Testing these hypotheses demonstrates the utility of this integrated electrochemical-mass spectrometric approach for elucidating redox-induced molecular transformations in complex natural organic matter and provides a promising avenue to identify potential new molecular proxies of DOM redox activity in peatlands.

## Materials and methods

### Sample collection

DOM samples were collected from three peat systems with high DOM concentrations (37–43 mg L⁻^1^). Samples are referred to as SO (Sosa), GT (Goethemoor), and KR (Krycklan, cf. Table S1). The SO sample was collected in a tributary of the Sosa drinking water reservoir (Ore Mountains of Saxony, Germany). The tributary, Neudecker Bach, is draining an area that features a mosaic of peatland, degraded peat, and forested slopes (50° 28′ 17.7″ N, 12° 40′ 14.3″ E) [34]. GT was collected from Goethemoor, a bog located near the summit of Brocken Mountain in the Harz Mountains, Germany (51° 47′ 23″ N, 10° 36′ 6″ E) [35]. KR originated from an upstream area of the Krycklan Catchment in northern Sweden, about 50 km northwest of Umeå (64° 25′ 0″ N, 19° 46′ 30″ E), representing the outlet of a fen (Kallkäls Mire) [36]. These peatlands differ in environmental conditions, allowing assessment of how site-specific factors influence DOM composition and redox characteristics. Water samples were collected in pre-combusted glass bottles (400 °C for 4 h), filtered using 0.45-µm regenerated cellulose membrane filters (Labsolute, Th. Geyer, Stuttgart, Germany) and stored in the dark at 4 °C until further analysis.

### Chemicals

Ultrapure water was obtained from a Milli-Q system (Merck, Darmstadt, Germany). Suwannee River fulvic acid (SRFA III; 3S101F) was purchased from the International Humic Substances Society. Reagents included sodium hydroxide (NaOH, ≥ 99% purity), potassium chloride (KCl, ≥ 99.5% purity), potassium dihydrogen phosphate (KH₂PO₄, ≥ 99.5% purity), 2-hydroxy-1,4-naphthoquinone (HNQ (lawsone, C₁₀H₆O₃), > 97% purity), and 9,10-anthraquinone-2,6-disulfonic acid disodium salt (AQDS (C₁₄H₆Na₂O₈S₂), > 98%) and were purchased from Th. Geyer, Stuttgart, Germany.

### DOC concentration

Non-purgeable organic carbon (NPOC) concentrations were measured using a DIMATOC 2100 analyzer (DIMATEC Analysentechnik GmbH, Essen, Germany) following EN 1484. Data processing was performed using DIMAQS software (DIMATEC Analysentechnik GmbH).

### Electrochemical setup and reduction procedure

Electrochemical reduction was performed in a gas-tight, two-compartment bulk electrolysis cell (50 mL; REDOXME, Sweden) equipped with a three-electrode configuration consisting of a reticulated glassy carbon working electrode, a platinum counter electrode, and an aqueous Ag/AgCl reference electrode (Fig. S1). Measurements were conducted using a ROXY potentiostat (Antec, Netherlands) under chronoamperometric control. A detailed description of the electrochemical cell configuration and validation procedure is provided in Text S1.

Experiments were carried out in 0.2 M KH₂PO₄ buffer (pH 7.0). The electrolyte was purged with high-purity N₂ for 1 h prior to each experiment and maintained under a gentle N₂ flow during measurements to ensure oxygen-free conditions. Although peatlands typically exhibit acidic conditions, a circumneutral phosphate buffer was selected to ensure electrochemical stability and comparability with previous non-mediated electrochemical studies of natural organic matter [6, 37, 38].

The electrochemical setup was first validated using reference compounds (HNQ (216 mg L⁻^1^) and AQDS (123 mg L⁻^1^)) at their respective reduction potentials (− 0.39 and − 0.60 V vs Ag/AgCl). SRFA was prepared at 50 mg L^−1^, referring to SRFA mass concentration, and assessed at − 0.49 V to allow comparison under comparable conditions [18, 39]. The carbon-normalized electron-accepting capacity (EAC) calculation (cf. Text S2) was based on the IHSS reported carbon content and is provided in Text S3. Subsequently, peatland-derived dissolved organic matter samples were reduced at − 0.59 V vs. Ag/AgCl (3 M KCl). This potential falls within the established redox window of quinone-like and aromatic moieties in DOM (− 0.3 to − 0.6 V vs. Ag/AgCl). The selected potential enables efficient reduction of redox-active functional groups while avoiding hydrogen evolution side reactions, consistent with previously validated non-mediated electrochemical approaches [39]. This potential was used to quantify non-mediated electron-accepting capacity and was not intended to selectively target one specific functional group.

The current response was recorded as a function of time, and EAC was determined by integrating the baseline-corrected current–time curve. To introduce DOM into the electrochemical cell, 5 mL of the DOM stock was added using a gas-tight syringe to pre-degassed 29 mL of KH₂PO₄ buffer, yielding final DOM concentrations between 5 mg C L⁻^1^ for SO and 7 mg C L⁻^1^ for GT and KR samples within the electrolytic cell. Completion of reduction was indicated by stabilization of the current signal [20]. Current–time (*i*–*t*) data were smoothed for visualization (Text S4).

For molecular characterization, 500 µL aliquots were collected immediately after DOM addition (*T*₀) and after current stabilization (*T*₁). All aliquots were transferred under N₂ into pre-purged LC–MS vials using a gas-tight syringe to prevent re-oxidation prior to LC-FT-ICR MS analysis. Further details on baseline definition, potential selection rationale, and sample handling procedures are provided in Text S1.

### Liquid chromatography (LC) and FT-ICR MS

DOM was separated using reversed-phase liquid chromatography (LC) as described before [29, 30]. A C18 column was used, with a water–methanol gradient (0.1% formic acid). FT-ICR mass spectrometry was performed on a solariX XR FT-ICR-MS (Bruker Daltonics, Billerica, MA, USA) equipped with a dynamically harmonized ICR cell and a 12 Tesla actively shielded superconducting magnet (Bruker BioSpin, Wissembourg, France). Ionization was achieved using an Apollo II electrospray ionization (ESI) source operating in negative ion mode. The ESI parameters were optimized as follows: capillary voltage 4.2 kV, nebulizer gas pressure 1.0 bar, drying gas 4.0 L/min at 200 °C. Each sample was analyzed in duplicate to ensure reproducibility. The ion accumulation time for each scan was 250 ms.

### Data processing

Chromatograms were segmented into twelve consecutive 1-min retention time (RT) intervals spanning 9–21 min after injection. MS data acquisition was initiated 8 min post-injection to exclude the early-eluting phosphate buffer fraction from the electrospray ionization source. This procedure reduced salt-induced ion suppression and minimized contamination of the ion source. Within each RT segment, mass spectra were averaged, and peaks with a signal-to-noise ratio ≥ 4 were selected. Calibration (m/z 150–1000) was performed using internal reference ions using the Lambda-Miner. Molecular formulas were assigned with elemental constraints C₁–₆₀, H₁–₁₂₂, O₀–₄₀, N₀–₂, S₀–₁ [40, 41] and filtered for chemical plausibility using molecular H/C, O/C, and N/C ratios, DBE, and DBE–O criteria [42, 43]. Peaks detected in ultrapure water or buffer blanks were excluded, and only formulas consistently observed in both sample replicates were retained.

Relative intensity changes between untreated (*T*₀) and electrochemically reduced (*T*₁) samples were evaluated using log₂(*T*₁/*T*₀) ratios calculated from base peak–normalized intensities for individual molecular formulas or the total ion chromatogram (TIC) for the segments spanning 9–21 min. For each common formula, intensity changes were calculated as log_2_(*T*_1_/*T*_0_) using base peak intensities. To evaluate whether formula-level intensity changes were stronger or weaker than the overall TIC response, the classification threshold was adjusted for the TIC change log_2_[(*T*_1_/*T*_0_)_TIC_]. A ± 1.5-fold change threshold (|log₂(*T*₁/*T*₀)|≥ 0.585) was applied to classify formulas as decreased, increased, or unchanged. This threshold exceeds measurement variability associated with ultrahigh-resolution mass spectrometric peak assignment and relative intensity normalization while remaining sensitive to systematic compositional shifts in complex DOM mixtures [44].

## Results and discussion

### Validation of the direct electrochemical reduction approach

**Fig. 1:**
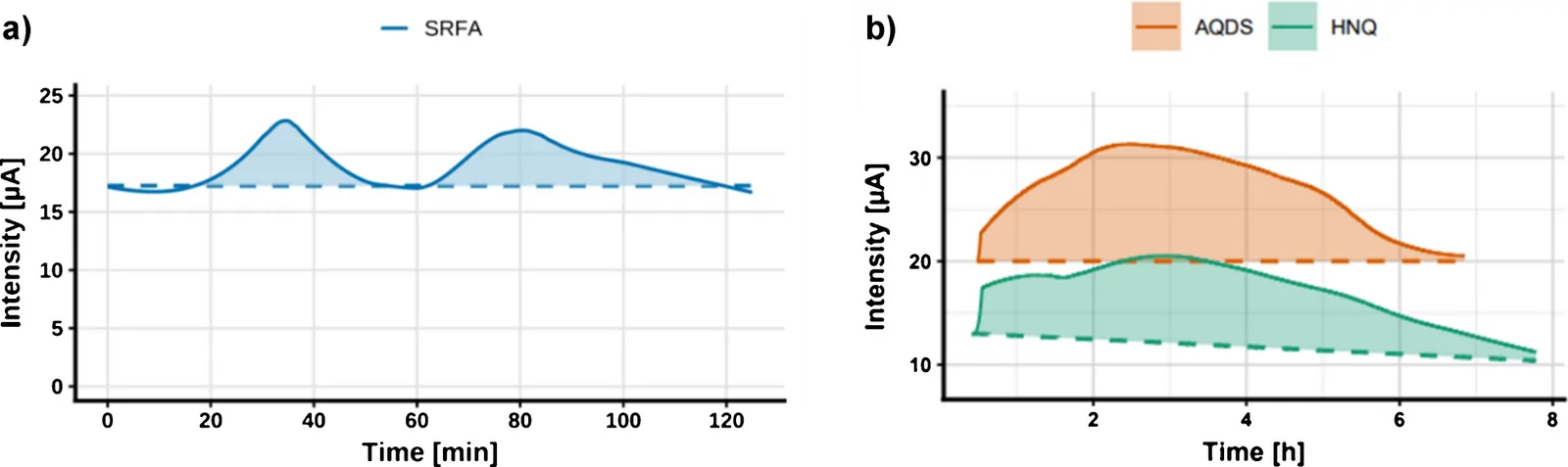
**a** Chronoamperometric (*i*–*t*) response of SRFA (50 mg L⁻^1^, blue, − 0.49 V vs. Ag/AgCl), showing two successive current peaks resulting from two sequential SRFA injections, and **b** chronoamperometric responses of AQDS (123 mg L⁻^1^, orange, − 0.60 V vs. Ag/AgCl), and HNQ (216 mg L⁻^1^, green, − 0.39 V vs. Ag/AgCl), raw amperometric traces shown in Fig. S2. Electron-accepting capacities (EACs) were determined by integrating the baseline-corrected current–time curves (dotted lines indicate the baseline)

To evaluate the performance of the DER system, SRFA (50 mg L⁻^1^) and the model quinones HNQ (216 mg L⁻^1^) and AQDS (123 mg L⁻^1^) were used as reference compounds (Fig. 1). Integration of duplicate current–time responses yielded EAC values of 319 and 279 µmol e⁻ (gC)⁻^1^, resulting in an average SRFA EAC of 299 ± 28 µmol e⁻ (gC)⁻^1^. These values are consistent with previously reported EACs for SRFA measured by direct, single-potential electrochemical reduction and correspond to approximately 60% lower values than those obtained by mediated electrochemical approaches [45], which access a broader redox window and therefore integrate a larger fraction of redox-active sites [18, 39, 46]. The chronoamperometric response of SRFA exhibited two distinct reduction features with peak durations of approximately 35 and 45 min; these two features resulted from two consecutive SRFA additions to evaluate reproducibility.

In contrast, HNQ and AQDS produced reduction responses that were broadened on a timescale of hours (approximately 6.9 h for HNQ and 5.8 h for AQDS). Time-integrated EAC values were 2121 µmol e⁻ (gC)⁻^1^ for HNQ and 6770 µmol e⁻ (gC)⁻^1^ for AQDS, indicating that small quinones can provide a substantial fraction of their stoichiometric electron-accepting capacity under direct electrochemical reduction conditions [18]. This behavior is consistent with discrete quinone/hydroquinone redox couples with well-defined electron-transfer stoichiometries. For comparison with theoretical limits assuming a two-electron quinone/hydroquinone redox couple, HNQ has a nominal maximum EAC of 16,700 µmol e⁻ (gC)⁻^1^, of which the measured value represents approximately 13%. AQDS has a maximum of 11,900 µmol e⁻ (gC)⁻^1^, and the experimentally determined value of 6770 µmol e⁻ (gC)⁻^1^ corresponds to approximately 50% of the theoretical EAC. Since the quinones were not pre-oxidized, the reduction starts from an equilibrium distribution of redox states rather than from fully oxidized quinones, and the full theoretical EAC cannot be accessed. Only the fraction present in the oxidized form contributes to the measured EAC. Since HNQ has a less negative standard potential than AQDS, the equilibrium at the initial electrode potential is shifted further toward reduced forms for HNQ, leaving a smaller oxidized fraction available for reduction. Thus, the measurable capacity is primarily governed by redox equilibrium, with mass transport and electron-transfer kinetics imposing additional constraints during bulk electrolysis.

In non-mediated bulk electrolysis, reduction is limited by diffusion of electroactive species to the electrode surface and by heterogeneous electron-transfer kinetics; complete electrochemical reduction therefore requires sufficient time for transport and extensive interfacial reaction of all reducible species [47]. Previous studies of humic substances have similarly shown that measured redox capacities depend on the applied potential and equilibration conditions [9, 18]. Accordingly, the EAC values obtained for SRFA reflect the fraction of redox-active moieties accessible under the applied conditions and timescale. Detailed EAC calculations for SRFA, HNQ, and AQDS are provided in Text S3.

#### Consistency of DOC concentration during the electrochemical reduction

We measured DOC concentrations before (*T*₀) and after (*T*₁) DER to verify whether DOC loss occurs during the electrochemical reduction. DOC concentrations decreased only slightly after DER (−6% for SO, −2% for GT, and −3% for KR), indicating no substantial bulk DOC removal. These small changes may reflect minor adsorption to electrode surfaces, sample handling, or analytical variability. Studies on the interaction of humic substances and DOM with electrode surfaces show that adsorption can occur, but its magnitude is often limited and depends strongly on solution chemistry and surface properties [48–50]. Such effects are therefore expected to occur mainly at the electrode-solution interface rather than causing bulk DOC loss. Furthermore, at the applied potentials, electron transfer is expected to involve redox-active functional groups, while mineralization to inorganic carbon is thermodynamically unfavorable [7, 19]. Accordingly, the results of our electrochemical treatment are best interpreted as changes in DOM redox state rather than substantial removal of organic carbon.

### Electron-accepting capacity of DOM across sites

**Fig. 2:**
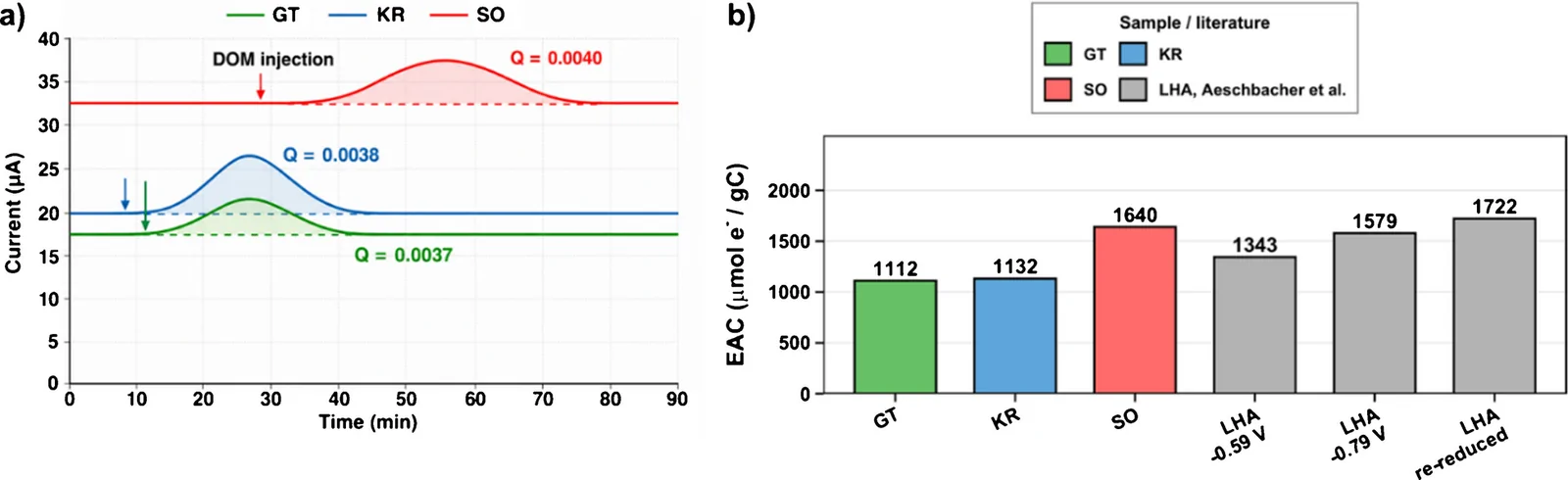
**a** Chronoamperometric (*i*–*t*) responses during direct electrochemical reduction of DOM from SO (5 mg C L⁻^1^, red), KR (7 mg C L⁻^1^, blue), and GT (7 mg C L⁻^1^, green) at −0.59 V vs. Ag/AgCl in phosphate-buffer electrolyte. DOM injection is indicated by arrows, and shaded areas represent the baseline-corrected integrated current used to calculate EAC. Current traces are smoothed for visualization (Text S4). **b** Comparison of EAC values obtained in this study with literature values reported for leonardite humic acid (LHA) measured by direct electrochemical reduction under different conditions by Aeschbacher et al. [18]

Amperometric analyses of DOM from the three peatland sites revealed clear differences in electrochemical behavior (Fig. 2). Integration of *i*–*t* curves yielded EACs of 1112 µmol e⁻ (gC)⁻^1^ for GT, 1132 µmol e⁻ (gC)⁻^1^ for KR, and a higher value of 1640 µmol e⁻ (gC)⁻^1^ for SO (raw amperometric traces shown in Fig. S3; calculation procedure described in Text S5), indicating a higher density of redox-active functional groups in SO as compared to GT and KR.

The EAC observed for the three DOM samples is generally consistent with the redox-active properties of DOM in peatlands, which may contain quinone, phenolic, and sulfur-containing moieties [32, 51]. The higher baseline current observed for SO may reflect sample-specific electrochemical interactions or ionic characteristics rather than non-faradaic charging, which would be expected to influence all samples similarly.

The magnitude of the EAC values measured here falls well within the range reported for humic substances and other redox-active organic materials when expressed on a carbon-normalized basis. Direct electrochemical reduction studies have reported EACs of approximately 1300–1700 µmol e⁻ (gC)⁻^1^ for leonardite humic acid (Fig. 2b, [18]), while plant-derived biochars containing quinone-like functionalities exhibit EACs of up to ~ 2000 µmol e⁻ (gC)⁻^1^ [52]. Other peat humic materials have EACs on the order of 10^3^–10^4^ µmol e⁻ (gC)⁻^1^, depending on redox state and analytical approach [51] (Fig. S4).

Accordingly, the EAC magnitudes observed for GT, KR, and SO are consistent with values expected for peat-derived DOM and support the presence of abundant redox-active functional groups known to govern electron-accepting behavior in peat organic matter [53]. These findings reinforce prior evidence that peatlands host DOM pools with a substantial capacity to participate in biogeochemical electron-transfer processes.

### LC-FT-ICR MS signatures of reduced DOM

**Fig. 3:**
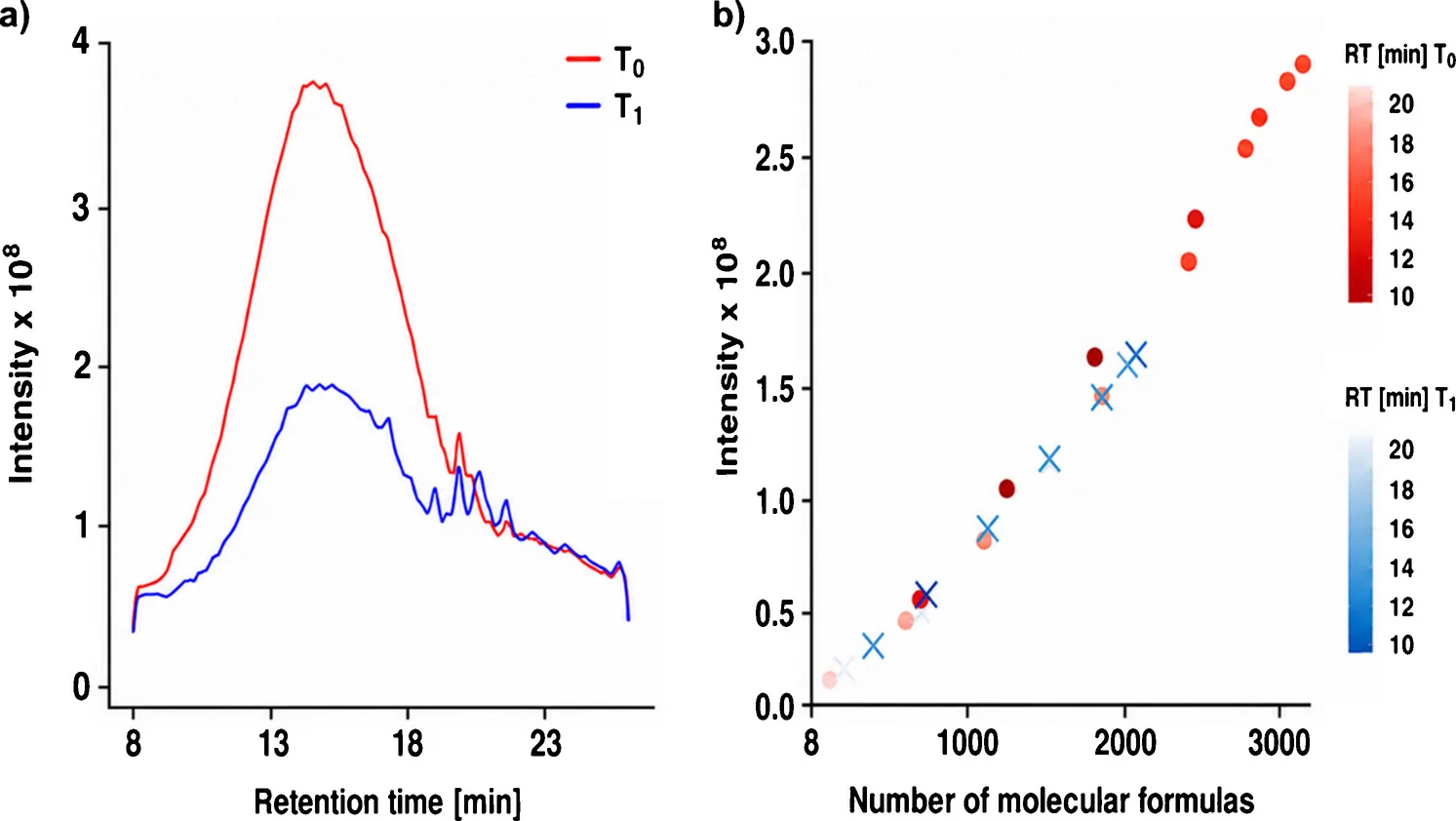
**a** Total Ion chromatograms (TIC) profiles of the KR sample at two time points (*T*₀ in red, *T*₁ in blue). The curves, showing summed ion intensity versus chromatographic retention time, indicate a clear overall decrease in signal after electrochemical reduction. **b** Scatter plot of summed intensity versus number of molecular formulas per retention-time bin (9–20 min), with colors representing chromatographic segments (red gradient = *T*₀, blue gradient = *T*₁)

FT-ICR MS analysis revealed a pronounced decrease in total ion chromatogram (TIC) intensity for the KR sample following DER (Fig. 3a), which was also apparent for the other DOM samples studied herein (Fig. S5). Duplicate injections yielded more than 40% decrease in summed intensity following electrochemical reduction. To evaluate whether this decline was also reflected on the level of accessible molecular information, the summed intensity of assigned molecular formulas was evaluated, also indicating a similar decrease (−44%) following DER (Fig. S6). The close agreement between summed intensity of assigned molecular formulas and TIC reductions suggests that the observed decrease reflects a true decline in assignable ion intensity rather than variability in chromatographic integration or ionization artifacts. Across the full dataset (KR, SO, and GT), the mean decrease in summed ion intensity was 30% (*n* = 3). This finding supports our first hypothesis that electrochemical reduction induces measurable changes in the LC-FT-ICR MS signal response of DOM.

Comparison of TIC values with the number of assigned molecular formulas (Fig. 3b) revealed a parallel decline. This pattern likely reflects the overall reduced signal intensity rather than a loss of molecular diversity, similar to the lower number of detected formulas in the very early LC segments with overall low intensity. Many mass peak features fall below the S/N threshold after reduction, and the DOM appears less complex. The similar chromatographic elution patterns observed for *T*₀ and *T*₁ suggest that electrochemical reduction did not induce substantial compositional shifts which would be reflected in altered polarity and thus retention time distribution. Instead, the uniform decrease in ion intensity suggests that molecule polarity (here, RP-LC elution behavior) did not indicate electrochemically accessible functional groups (Fig. 3a).

SRFA only showed a 16% reduction in TIC after DER at, however, a lower potential of − 0.34 V vs. Ag/AgCl (Fig. S7). As an IHSS reference material representing the XAD-8–isolated hydrophobic fulvic acid fraction, SRFA is compositionally less heterogeneous than the non-extracted peat DOM analyzed here [54]. Consistent with previous FT-ICR MS studies showing SRFA to be predominantly CHO-rich and less structurally diverse than environmental DOM [55], the lower TIC decrease suggests a lower proportion of functional groups available for reduction. In contrast, the peat-derived DOM exhibited stronger intensity shifts, consistent with broader molecular diversity and a larger pool of redox-active moieties (Text S6).

Overall, despite minimal changes in bulk DOC concentration, the substantial decreases in TIC intensities demonstrate that electrochemical reduction induces significant molecular-level transformations that result in a lower ionization efficiency of the reduced DOM at stable carbon concentration. The combined analytical approach (DER/LC-FT-ICR MS) therefore enables evaluation of and new insights into redox-driven transformations in natural organic matter.

### Molecular signatures of DOM reduction

**Fig. 4:**
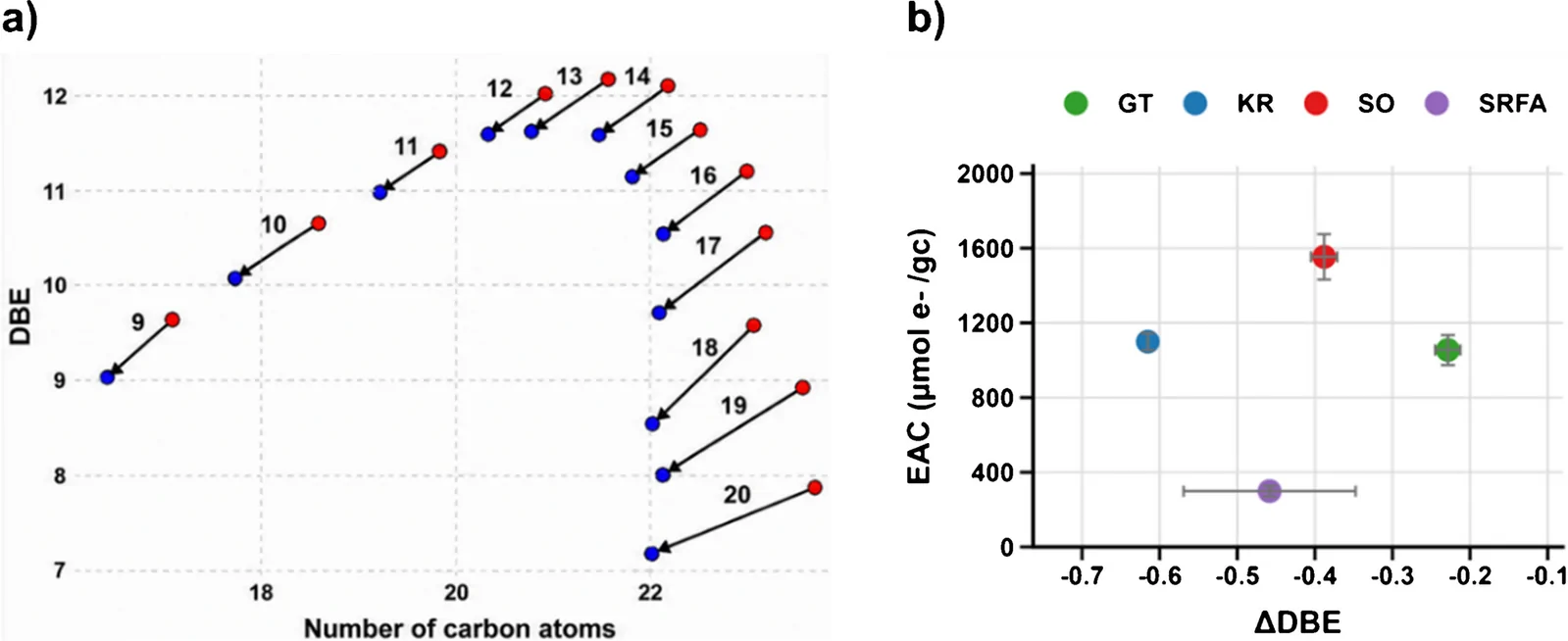
**a** Intensity-weighted average double bond equivalents (DBE) as a function of carbon number derived from LC-FT-ICR MS data for *T*₀ (red) and *T*₁ (blue) of the KR sample. Arrows denote the direction of compositional shifts from *T*₀ to *T*₁. Numbers on arrows (9–20) correspond to retention-time intervals. The analysis includes all molecular formulas assigned for the KR sample during electrochemical reduction. Corresponding figures for molecular H/C and O/C and limited to molecular formulas consistently or uniquely detected in both *T*₀ and *T*₁ are shown in Figs. S9 and S10. **b** Relationship between ΔDBE derived from aggregated intensity-weighted-average LC-FT-ICR MS molecular formula data and bulk electron-accepting capacity (EAC) determined by DER for SRFA and peat-derived DOM samples (GT, KR, SO). Each circle represents the mean value of duplicate measurements for one sample; horizontal and vertical error bars indicate the duplicate ranges for ΔDBE and EAC, respectively

DER produced consistent changes in the LC-FT-ICR MS molecular signal distribution across all chromatographic retention time segments of the DOM samples, as shown by shifts in intensity-weighted average DBE toward lower values (−0.9 to −1.2, Fig. 4a, Table S2). Comparable DBE decreases were observed for the SO sample (Fig. S8). These shifts were observed across the elution profile, indicating that the reduction-related response was not restricted to a single retention time or polarity fraction. However, the apparent DBE shifts should not be interpreted as direct evidence of DBE reduction of every molecular formula across the segments. Instead, it reflects a combined response of changes in relative LC-FT-ICR MS signal intensities and changes in the detected formula set (e.g., via double bond saturation). The shared formula analysis therefore provides an important complementary view, showing that a substantial part of the DBE response is related to intensity redistribution within formulas detected in both *T*_0_ and *T*_1_, rather than to a pronounced change in molecular formula composition. Consistent with the DBE trend (Fig. 4a), DBE/C values were lower after DER across most retention time segments, with a more apparent decrease when all formulas were considered and a smaller but consistent decrease among common formulas between *T*_0_ and *T*_1_ (Fig. S11). This supports a shift toward lower unsaturation relative to carbon number and supports a rather subtle formula-level signal redistribution response rather than a major overall transformation of DOM composition.

The EAC-derived effective DBE change (ΔDBE_eff_) was calculated using an intensity-weighted mean number of carbon atoms per molecule (Text S7). ΔDBE_eff_ represents a theoretical value of average DBE decrease that could account for the measured electron uptake by carbon atoms in a DOM molecule. Assuming that a decrease of one double bond unit corresponds to the uptake of two electrons, the EAC values of approximately 1–1.6 × 10^3^ µmol e⁻ (gC)⁻^1^ for the DOM samples correspond to ΔDBE_eff_ values of approximately −0.14 to −0.20 (Text S7). The relatively small values indicate that the measured bulk electron uptake would only induce a limited average change in DBE per molecule, supporting the absence of extensive molecular composition changes.

We compared the measured non-mediated EAC with the independently calculated DBE change from LC-FT-ICR MS data (ΔDBE_MS_, Fig. 4b). KR showed the largest DBE_MS_ decrease, approximately −0.62, whereas SO and GT showed smaller decreases of approximately −0.39 and −0.23, respectively. SRFA showed an ΔDBE_MS_ of approximately −0.46 but had a lower EAC than the DOM samples. Notably, the relationship between EAC and ΔDBE of the four investigated samples did not show a clear dependency across samples. The larger magnitude of ΔDBE_MS_ as compared to the ΔDBE_eff_ also reflects changes in the LC-FT-ICR MS signal distribution in the detected formula set that may be influenced by ionization efficiency and structural isomerism.

Importantly, the relationship between EAC and ΔDBE_MS_ should not be interpreted as a direct quantification of reduced C = C bonds. Notably, C = O groups in, e.g., quinones, also contribute to DBE [56] and reducible functional groups at the applied potential. Accordingly, the observed ΔDBE decrease should therefore be interpreted as an intensity-weighted molecular signal response that is consistent with selective changes in redox-active, unsaturation-related molecular features rather than as evidence for extensive saturation of double bonds or aromatic frameworks. Similar selective behavior has been reported for DOM subjected to controlled electrochemical or photochemical reduction, where a small fraction of molecules accounts for most of the redox capacity [9, 57]. Factors such as the density, accessibility, and conjugation length of electrochemically active moieties likely govern how efficiently molecular-scale changes translate into bulk electron uptake, consistent with prior electrochemical characterizations of humic and fulvic substances [52].

**Fig. 5:**
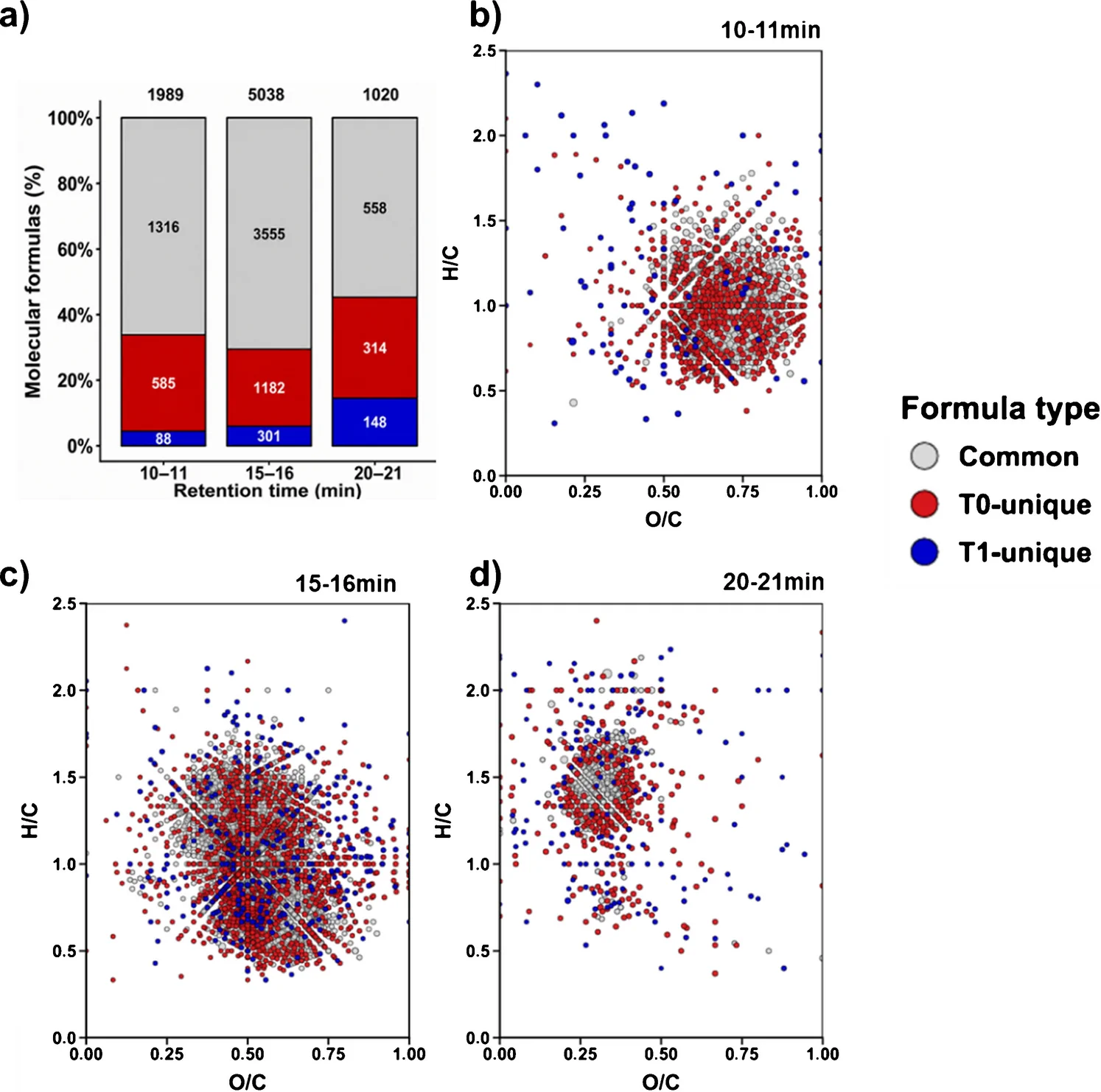
**a** Proportions of common and unique molecular formulas between *T*_0_ and *T*_1_ across three selected polarity fractions of the KR sample: more polar (10–11 min), mid-polar (15–16 min), and less polar (20–21 min). Common formulas were detected in both *T*_0_ and *T*_1_, whereas *T*_0_-unique and *T*_1_-unique formulas were detected only before and after DER, respectively. **b**–**d** Molecular H/C vs O/C ratios of molecular formulas detected in the corresponding polarity fractions. Point size represents the relative peak intensity

At the molecular formula level, the majority of formulas are shared between *T*_0_ and *T*_1_ across LC retention-time windows (≈55–71%; Fig. 5a), confirming that the overall molecular composition of peatland DOM remains largely preserved following DER. Although the relative contributions of *T*₀-unique (detected only prior to reduction), *T*₁-unique (detected only after reduction), and common formulas (detected at both time points) vary along the retention-time gradient, common formulas dominate throughout. In molecular H/C versus O/C space (Fig. 5b–d), *T*₁-unique formulas largely overlap with *T*₀-unique and common formulas, indicating that they remained within the pre-existing chemical space. This suggests compositional adjustment within the established molecular formula space rather than removal or formation of distinct compound classes.

**Fig. 6:**
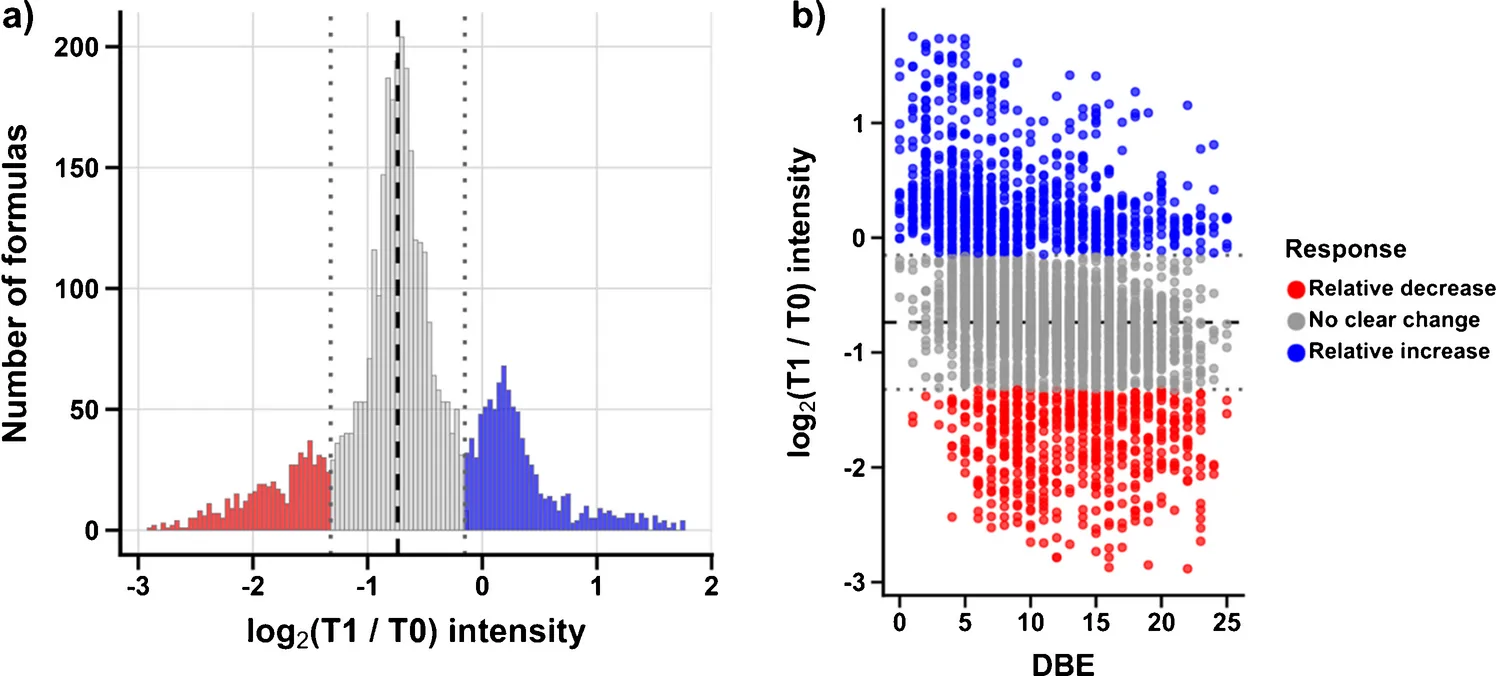
**a** Distribution of intensity ratios (using base peak normalized intensities) for molecular formulas detected in both untreated (*T*₀) and electrochemically reduced (*T*₁) KR samples, calculated as log_2_(*T*_1_/*T*_0_). The dashed line indicates log_2_(*T*_1_/*T*_0_), and dotted lines show the ± log_2_(1.5) threshold. Colors indicate formulas with a relative decrease compared to TIC response (red), no clear change within the threshold range (gray), or relative increase compared with the TIC response (blue). **b** Formula intensity ratios plotted against double bond equivalents (DBE), using the same molecular formulas and color classification as in **a**

Intensity-weighted averages of carbon number and DBE across *T*_0_-unique, *T*_1_-unique, and common molecular formulas (Table S3) provide additional compositional context and are consistent with the trends observed for the total molecular composition (Fig. 4a) and for the common formulas (Fig. 6b). Across the examined LC retention-time windows, the unique formulas followed the same trend observed for the common and all molecular formulas, indicating a pronounced shift toward lower DBE values and lower carbon numbers after DER (Fig. S10). Together with the observed decrease in TIC after DER (Fig. 3a), these results are consistent with a preferential decrease in MS signal response of larger and more unsaturated molecular formulas, rather than with the formation of a systematically distinct molecular composition. This apparent signal decrease may reflect electrochemical transformation, changes in ionization efficiency, adsorption, or a combination of these processes, and therefore cannot be assigned to a single loss mechanism. Although electrochemical follow-up reactions are conceivable in principle [58], the data align more closely with previous electrochemical studies of humic substances reporting shifts in oxidation state without evidence for extensive molecular restructuring [15, 18, 52]. Overall, DER induces measurable but limited shifts within the existing molecular space without generating a distinct new compositional domain.

### Molecular drivers of DER activity within DOM

DER reduced the TIC and number of formulas detected at similar injected amounts of DOC, making comparisons of reduction effects between samples potentially confounded by a reduction in ionization efficiency. To distinguish whether the DBE shift observed for all assigned formulas mainly reflected changes in molecular formula-level composition or changes in formula-level intensities, the response was further evaluated for formulas shared between *T*_0_ and *T*_1_ samples (Fig. 6).

The molecular response of the KR sample was characterized by a shift toward lower relative peak intensities for molecular formulas detected in both *T*₀ and *T*₁ (Fig. 6a). Overall, 81.2% of the common formulas showed negative log_2_(*T*_1_/*T*_0_) values after reduction. To account for the overall TIC decrease, the classification threshold was shifted from zero to the average TIC response (Fig. 3a). Accordingly, formulas were classified as showing a stronger, similar, or weaker intensity decrease relative to the overall TIC decrease. This analysis indicates that part of DER response occurred through redistribution of signal intensities among formulas shared by *T*_0_ and *T*_1_, rather than only through changes in formula detection. Thus, Fig. 6 complements Fig. 4a by distinguishing intensity redistribution within the shared formula set from changes in the full detected formula pool.

When related to molecular descriptors, the intensity response showed the clearest association with double bond equivalents (DBE) (Fig. 6b). A weak but significant monotonic association was observed between log_2_(*T*_1_/*T*_0_) intensity ratios and DBE (Spearman’s *ρ* =  − 0.354, *p* < 0.001; *R*^2^ = 0.108), indicating that DBE alone explains only a limited fraction of formula-level intensity response. Nevertheless, the distribution suggests a separation around DBE ≈ 10 rather than a continuous gradient. Group-wise comparison confirmed that formulas with DBE > 10 exhibited more negative log₂(*T*₁/*T*₀) values than those with DBE ≤ 10 (mean − 0.792, median − 0.783, *n* = 2076 vs mean − 0.457, median − 0.558, *n* = 2069; Wilcoxon rank-sum test *p* < 0.001) [59]. This indicates that the DER-related signal decrease was preferentially associated with formulas of higher DBE, while the low *R*^2^ shows that DBE is not sufficient as a single descriptor of the response.

In contrast, no strong systematic dependence of log₂(*T*₁/*T*₀) on O/C ratio was observed (Fig. S12c). Intensity decreases occurred across the full O/C range without evidence for a distinct threshold or monotonic trend, suggesting that overall oxygen abundance alone does not control the electrochemical response. The weaker relationship with DBE–O compared to DBE (Fig. S12b) further indicates that accounting for oxygen content did not improve explaining the intensity response after DER. Rather, DER appeared to affect molecular formulas where unsaturation and oxygen-containing functionalities occur in specific structural environments that cannot be resolved from molecular formulas alone [60]. AI_mod_ likewise only showed a weak relationship with intensity change (Fig. S12d). Together, the descriptor-based comparison suggests that DER response was associated with unsaturation-related formula properties, but cannot be explained by oxygen content, aromaticity, or any single formula-derived descriptor alone. Our results are consistent with Kurek et al. [20], who showed that redox-active DOM spans a broad compositional range, including highly unsaturated formulas with relatively low O/C.

Altogether, our results support the second hypothesis that DER-induced intensity changes are molecularly selective rather than uniformly distributed across DOM. The decrease in intensity-weighted average DBE after DER, together with formula-level response, supports that more unsaturated molecular features are more reactive toward reduction, particularly formulas with DBE values above approximately 10. However, because DBE, DBE-O, and AI_mod_ are formula-derived descriptors, they do not uniquely identify specific reactive functional groups [24, 56]. Notably, the current molecular formula-level data do not allow pinpointing to a quinone-specific reduction pattern but indicate redox-active molecular formulas that may serve as a basis to refine redox indicators and to investigate structural features [61].

## Conclusion

This study demonstrates that mild direct DER induces reproducible molecular-level changes in the LC-FT-ICR-MS signal distribution and that these shifts are associated with bulk electrochemical behavior. Across all samples, DER resulted in pronounced mass spectrometric intensity decreases, while DOC concentration changed only slightly, indicating no substantial bulk carbon loss. Thus, the observed signal decrease is best interpreted as a redox-induced change in relative signal intensities among molecular formulas.

DER did not cause a major overall transformation of the DOM formula space, but induced measurable and reproducible shifts within the existing molecular assemblage. Intensity-weighted average DBE values decreased consistently following DER, and ΔDBE was positively associated with bulk EAC. This association suggests a link between unsaturation-related molecular signals and bulk electron uptake under the applied electrochemical conditions. Evaluation of relative intensity changes among shared molecular formulas showed that higher-DBE formulas were preferentially affected by DER. Among the tested molecular descriptors, DBE showed the best association with relative intensity change, whereas DBE–O, AI_mod_, and O/C displayed weaker relationships. Together, these findings indicate that molecular unsaturation represents an important compositional descriptor associated with the electrochemical response, but not the only factor controlling formula-level intensity changes. Thus, the observed response likely reflects a broader unsaturation-related molecular signal response, including but not limited to quinone-like structures. However, LC-FT-ICR MS does not allow definitive assignment of the reduced functional groups or structural motifs transformed during DER. Complementary structural approaches, such as FTIR, NMR, tandem MS, or stable isotope labeling/H–D exchange experiments, would therefore be required to identify the specific functional groups and pathways involved.

An open question is how the molecular signatures generated under controlled DER compare with those arising from natural reduction pathways in peatland environments, where electron transfer is governed by microbially mediated and mineral-associated redox processes rather than electrochemical control. Future studies should examine whether the reduction patterns observed here persist or transform under natural anoxic conditions. Such work would clarify the extent to which DER represents a mechanistic analog for redox-driven transformations in complex peatland DOM.

## Supplementary Information

Below is the link to the electronic supplementary material.

Supplementary file1 (DOCX 2.75 MB)

## Data Availability

Processed and quality-checked data for all samples and segments and the final list of molecular formulas with direct electrochemical reduction are available from the UFZ Data Investigation Portal: https://doi.org/10.48758/ufz.16838. Raw MS files can be shared upon request.
