# Supplementary material for: Molecular signatures of electrochemically reduced peatland dissolved organic matter resolved by liquid chromatography Fourier transform ion cyclotron resonance mass spectrometry
Source: Anal Bioanal Chem. 2026 Jul 17;418(19):6441–53. doi: 10.1007/s00216-026-06668-y (PMC13585793; doi:10.1007/s00216-026-06668-y)
Supplement: Supplementary file 1 [file 216_2026_6668_MOESM1_ESM.docx]

Supporting information to

**Molecular signatures of electrochemically reduced peatland dissolved organic matter resolved by liquid chromatography Fourier transform ion cyclotron resonance mass spectrometry**

Rania Mobarak^1^, Carsten Simon^1^, Patrick Guth^2^, Klaus Holger Knorr^2^, Maximilian P. Lau^3*^, Oliver J. Lechtenfeld^1*^

1 BioGeoOmics, Department of Environmental Analytical Chemistry, Helmholtz Centre for Environmental Research-UFZ, 04318 Leipzig, Germany.
2 Institute of Landscape Ecology, Ecohydrology & Biogeochemistry Group. University of Münster, 48149 Münster, Germany
3 Interdisciplinary Environmental Research Centre, Technische Universität Bergakademie Freiberg, 09599 Freiberg, Germany

*corresponding authors: Maximilian P. Lau (maximilian.lau@mineral.tu-freiberg.de), Oliver J. Lechtenfeld (oliver.lechtenfeld@ufz.de)

No. of pages: 17

No. of tables: 3

No. of figures: 12

Contents

[Text S1: Detailed Electrochemical Setup, Validation, and Data Processing	3](#_Toc231679569)

[Text S2: Calculation of electron-accepting capacity (EAC)	4](#_Toc231679570)

[Text S3: Calculation of Electron Accepting Capacities (EAC) for SRFA, HNQ, and AQDS Samples	5](#_Toc231679571)

[Text S4: Smoothing of current–time traces for DOM samples (GT, KR, SO)	6](#_Toc231679572)

[Text S5: Calculation of Electron Accepting Capacities (EAC) for dissolved organic matter (DOM) Samples	7](#_Toc231679573)

[Text S6: Compositional Characteristics and Redox Response of SRFA Compared to Peat-Derived DOM	7](#_Toc231679574)

[Text S7: Calculation of Effective ΔDBE Equivalent to Measured EAC for the KR Sample	8](#_Toc231679575)

[*Table S1: Description of sampling sites in this study.*	8](#_Toc231679576)

[*Table S2: Intensity weighted-average (wa) carbon number and double bond equivalents (DBE), calculated for all molecular formulas in the KR-DOM sample, for untreated (T₀) and reduced (T₁) DOM across retention time (RT) segments and corresponding differences (Δ).*	10](#_Toc231679577)

[*Table S3: Intensity-weighted average (wa) carbon number and double bond equivalents (DBE) for T_0_-unique, T_1_-unique, and common molecular formulas in the KR-DOM samples across selected retention-time windows (cf. Figure 5 in main text).*	10](#_Toc231679578)

[*Figure S1: Schematic of the two-compartment bulk electrolysis cell (50 mL) used for DER experiments. The working electrode (WE) and counter electrode (CE) compartments are separated by a frit and equipped with gas inlet/outlet ports. Adapted from Redoxme AB (Sweden).*	11](#_Toc231679579)

[*Figure S2: Chronoamperometric (i–t) response of AQDS (123 mg L⁻¹) and HNQ (216 mg L⁻¹). Raw current–time data are shown in grey, and smoothed traces are shown in orange (AQDS, −0.60 V vs Ag/AgCl) and green (HNQ, −0.39 V vs Ag/AgCl). Electron-accepting capacities (EACs) were determined by integration of the baseline-corrected i–t curves; dotted lines indicate the baseline.*	11](#_Toc231679580)

[*Figure S3: Representative chronoamperometric profiles (raw data) for GT, SO, and KR samples.*	12](#_Toc231679581)

[*Figure S4: Comparison of electron accepting capacities (EAC) of DOM from this study with literature data. EAC values of DOM samples from this study were determined by direct electrochemical reduction and compared with mediated EAC values reported in the literature references [1, 2, 10, 11]*	12](#_Toc231679582)

[*Figure S5: (a) Total ion chromatograms (TIC) profiles of the SO-DOM sample at two time points (T₀ in red, T₁ in blue). The curves, representing summed ion intensity as a function of chromatographic retention time, show a clear overall decrease in signal after DER. (b) Total ion chromatograms (TIC) profiles of the GT-DOM sample at two time points (T₀ in red, T₁ in blue). The summed ion intensity versus retention time likewise indicates a noticeable decrease in overall signal following electrochemical reduction.*	13](#_Toc231679583)

[*Figure S6: Total assigned chromatograms (TACs) of KR-DOM sample at two time points (T_0_, red; T_1_, blue), plotted as peak intensity versus chromatographic time.*	13](#_Toc231679584)

[*Figure S7: Total ion chromatograms (TICs) of Suwannee River Fulvic Acid (SRFA) at two time points: before reduction (T₀, red) and after reduction (T₁, blue).*	14](#_Toc231679585)

[*Figure S8: Intensity-weighted average double bond equivalent (DBE) versus number of carbon atoms derived for SO-DOM sample from LC-FT-ICR-MS data for T₀ (red circles) and T₁ (blue circles). Arrows indicate the direction of compositional change from T₀ to T₁. labels indicate the corresponding 1-min retention time from 10-11 to 21-22 min*	14](#_Toc231679586)

[*Figure S9: Intensity-weighted average H/C versus O/C for common formula between T_0_ and T_1_ derived from LC-FT-ICR MS data for KR-DOM sample. Segment numbers (1–12) correspond to 1-min retention-time intervals (e.g., 1 = 9–10 min, 2 = 10–11 min, … 12 = 20–21 min).*	15](#_Toc231679587)

[*Figure S10: Intensity-weighted average double bond equivalents (DBE) versus carbon number derived from LC-FT-ICR MS data for the KR sample. (a) Common molecular formulas detected in both T₀ (red) and T₁ (blue). Arrows indicate the direction of compositional change from T₀ to T₁ within each retention-time segment. (b) Unique molecular formulas detected in T₀ (red) or T₁ (blue). Segment numbers (1–12) correspond to 1-min retention-time intervals (e.g., 1 = 9–10 min, 2 = 10–11 min, … 12 = 20–21 min). Values represent intensity-weighted averages within each segment.*	15](#_Toc231679588)

[*Figure S11: Changes in DBE/C after DER for the KR-DOM sample. DBE/C values across LC retention time for T_0_ before DER and T_1_ after DER, calculated from (a) all detected formulas and (b) formulas common to both T_0_ and T_1_ within each retention time segment.*	16](#_Toc231679589)

[*Figure S12: (a) Log₂(T₁/T₀) intensity ratios (base peak–normalized) for molecular formulas detected in both untreated (T_0_) and electrochemically reduced (T_1_) KR-DOM, plotted against (a) H/C, (b) DBE-O, (c) O/C, and (d) AI_mod_. The dashed line indicates the TIC derived log_2_[(T_1_/T_0_) response, and dotted lines indicate the ±log_2_(1.5) threshold. Colors indicate formulas with a relative decrease compared with the TIC response (red), no clear change within the threshold range (gray), or relative increase (blue).*	16](#_Toc231679590)

[References	17](#_Toc231679591)

# Text S1: Detailed Electrochemical Setup, Validation, and Data Processing

**Electrochemical cell configuration**

A gas-tight, two-compartment bulk electrolytic cell (50 mL, REDOXME, Norrköping, Sweden) constructed from inert glass and PEEK materials was used (Fig. S1). The working electrode was a reticulated glassy carbon electrode (OD 30 mm, ID 20 mm, height 25 mm, platinum wire contact), providing a high surface area-to-volume ratio. The counter electrode was a platinum wire (50HX15, 0.6/250 mm, 99.9% purity, gold-plated pin), and the reference electrode was an aqueous Ag/AgCl electrode.

**Electrolyte composition and rationale**

Electrochemical reduction was conducted in 0.2 M potassium dihydrogen phosphate (KH₂PO₄, pH 7.0), prepared according to established protocols [1, 2]. The buffer ensured sufficient ionic conductivity, minimized artifacts due to proton accumulation or depletion during electron transfer, and provided reproducible experimental conditions. Although peatlands typically exhibit acidic conditions (pH 4–5), a neutral phosphate buffer was selected to ensure electrochemical stability and comparability with previous electrochemical studies.

**Validation with reference compounds**

System validation was performed using SRFA, HNQ, and AQDS introduced in their equilibrium state (no pre-oxidation). The electrochemical cell was equilibrated at the respective reduction potential prior to injection of each standard compound.

**Baseline definition and data processing**

Baseline current was defined as the stable current recorded in buffer prior to DOM addition. electron-accepting capacity (EAC) was determined by integrating the area under the baseline-corrected current–time curve. Current–time (i–t) traces were smoothed for visualization purposes (Text S4).

**Oxygen exclusion and sample handling**

The electrolyte was purged with high-purity N₂ (0.5) for 1 h prior to each experiment, and a low N₂ flow was maintained throughout measurements to prevent oxygen intrusion. For molecular analysis, aliquots were transferred using a gas-tight metal syringe into 2 ml glass vials pre-purged with nitrogen, allowing injection through septa without exposure to ambient air.

**Statistical analysis of descriptor-dependent intensity changes**

Differences in log₂(T₁/T₀) distributions between DBE classes (DBE > 10 vs DBE ≤ 10) were evaluated using a two-sided Wilcoxon rank-sum test because log₂ intensity ratios were non-normally distributed [3].

# Text S2: Calculation of electron-accepting capacity (EAC)

Here we describe how electron-accepting capacity (EAC) was derived from electrochemical (EC) measurements. Current–time curves obtained during amperometric analysis were integrated to determine the total charge transferred during reduction. Peak areas were integrated using Origin software (OriginLab), yielding the total charge (Q) according to:

**(1)** $Q=I\times t$

where Q is the charge (C), I is the measured current (A), and t is time (s).

The number of transferred electrons (nₑ_⁻_) was then calculated from the measured charge using Faraday’s law:

**(2)** $n_{e-} =\frac{Q}{F}$

where F is the Faraday constant (96,485 C mol⁻¹).

To normalize electron transfer to carbon content, the mass of dissolved organic carbon (m_c_) in the electrochemical cell was calculated as:

**(3)** m_c_ = C_DOC_ · V

where m_c_ is mass of carbon (g), C_DOC_ is the dissolved organic carbon concentration (g L⁻¹) and V is the solution volume (L).

Electron-accepting capacity (EAC) was then calculated by dividing the moles of transferred electrons by the mass of carbon:

**(4)** $EAC=\frac{n_{e-}}{m_{c}}$

yielding EAC in mol e⁻ gC⁻¹.

For reporting purposes, values were converted to micromoles:

**(5)** EAC (μmol e⁻ /gC) = EAC (mol e⁻ /gC) × 10⁶.

# Text S3: Calculation of Electron Accepting Capacities (EAC) for SRFA, HNQ, and AQDS Samples

**SRFA**

**1^st^ peak**

Amount of carbon in SRFA from IHSS = 53.30 %

Area under peak = charge in coulomb = amp x sec, Q= 66.55 × 10^-6^ × 60 = 3.99×10^−3^ C

Number of transferred electrons = Q / Faraday constant = 3.99×10^−3^ / 96485 = 4.14×10^−8^ mol e^−^

Mass of carbon in SRFA = 0.05 g/L × 0.005 L × 0.533 = 0.00013 gC

EAC in moles e^-^/gC SRFA = 4.14 × 10 ^-8^/ 0.00013 gC= 3.19×10^−4^ mol e^-^/gC = **319** μmol e^-^/ gC

**2^nd^ peak**

Amount of carbon in SRFA from IHSS = 53.30 %

Area under peak = charge in coulomb = amp x sec, Q= 58.5 * 10^-6^ × 60 = 3.51×10^−3^ C

Number of transferred electrons = Q/ Faraday constant = 3.51×10^−3^ /96485 = 3.6379×10^−8^ mol e^−^

Mass of carbon in SRFA

0.05 g/L × 0.005 L × 0.533 = 0.00013 gC

EAC in moles e^-^/gC SRFA =3.6379×10^−8^ mol e^-^/ 0.00013 gC = 2.7301×10^−4^ mol e^-^/gC = **273** μmol e^-^/ gC

**HNQ**

Area under peak = 2544 × 10^-6^ × 60 = 0.15264 C

Number of transferred electrons = 0.15264 C/ 96485 = 1.58 × 10^-6^ mol e^-^

HNQ (C10 H6 O3): it contains 10 carbons

Molar mass of HNQ = 174.148 g/mol

Fraction of carbon by mass in HNQ = 120.1 / 174.148 = 0.689

So, 68.9 % of mass of HNQ is carbon

Mass of carbon in HNQ in gram = conc of HNQ × carbon mass fraction × applied volume =

0.2165 g/L × 0.689 × 0.005 L = 7.458 × 10^-4^ g

EAC = number of transferred electrons / grams of carbon = 1.58 × 10^-6^ / 7.458 × 10^-4^ = 2.1212222 × 10^-3^ mol e^-^/gC

EAC in μmol e^-^/gC = 2121 μmol e^-^/gC

**AQDS**

Area under peak = 2733 × 10^-6^ × 60 = 0.16398 C

Number of transferred electrons = 0.16398 C/ 96485 = 1.6999 × 10-6 mol e^-^

Mass of carbon in AQDS in gram

Carbon fraction in AQDS

AQDS (disodium salt): C14H6Na2O8S2

Carbon mass per mole:

mC= 4×12.011= 68.15 gC/mol

Molar mass of AQDS:

M≈ 412.29 g/mol

Fraction of carbon by mass= 168.15 / 412.29 = 0.4078

So carbon fraction ≈ 0.408 (40.8%).

Mass of carbon of AQDS in gram = 0.123 g/L×0.408×0.005 L= 2.51×10^-4^ gC

EAC= 1.6999 × 10^-6^ mol e^-^ / 2.51×10^-4^ gC = 6.77×10^-3^ mol e^-^/ gC= 6770 μmole^-^ /gC

# Text S4: Smoothing of current–time traces for DOM samples (GT, KR, SO)

To improve visual clarity of the current–time profiles, raw electrochemical data were smoothed using locally weighted regression (LOESS). Comparison of the raw and smoothed traces indicates that a moderate smoothing strength was applied, sufficient to suppress high-frequency noise while preserving the timing, amplitude, and width of the DOM-injection-induced current peaks and maintaining stable pre- and post-injection baselines. Based on the degree of smoothing observed, the effective span corresponds to approximately 30–40 % of the data points, consistent with commonly used LOESS settings for electrochemical time-series visualization. Smoothing was applied only for graphical presentation, and all quantitative analyses were conducted using the unsmoothed raw data.

# Text S5: Calculation of Electron Accepting Capacities (EAC) for dissolved organic matter (DOM) Samples

**GT** sample (7 mg/L)

Area under peak = charge in coulomb = amp.sec, Q= 62 * 10^-6^ * 60 = 0.00372 C

Number of transferred electrons = Q/ Faraday constant = 0.00372/96485 = 3.85 × 10^-8^ mol e^-^

Mass of carbon in DOM in gram = 7 mg/L × 0.005 L = 0.035 mg C = 3.5 × 10^-5^ g C

EAC in moles e^-^/gC= 3.85 × 10^-8^ mol e^-^ / 3.5 * 10^-5^ g = 0.0011 mol e⁻/gC = **1100** µmol e⁻/gC

**KR** sample (7 mg/L)

Area under peak = charge in coulomb = amp.sec, Q= 64 * 10^-6^ × 60 = 0.00384 C

Number of transferred electrons = Q/Faraday constant = 0.00384 / 96485 = 3.98 * 10^-8^ mol e^-^

Mass of carbon in DOM in gram = 7 mg/L × 0.005 L = 0.035 mg C= 3.5 * 10^-5^ g C

EAC in moles e⁻/gC= 3.98 * 10^-8^ mol e^-^ / 3.5 * 10^-5^ g = 0.001137 mol e⁻/gC = **1137** µmol e⁻/gC

**SO** sample (5 mg/L)

Area under peak = charge in coulomb = amp.sec, Q = 66 × 10^-6^ * 60 = 0.00396 = 4 × 10^-3^ C

Number of transferred electrons = Q/ Faraday constant = 4 × 10^-3^ / 96485 = 4.1× 10^-8^ mol e^-^

Mass of carbon in DOM in gram = 5 mg/L × 0.005 L = 0.025 mg C = 2.5 × 10^-5^ g C

EAC in moles e⁻/gC = 4.1 * 10^-8^ mol e^-^ / 2.5 × 10^-5^ g = 1.64 × 10^-3^ mol e⁻/gC = **1640** µmol e⁻/gC

# Text S6: Compositional Characteristics and Redox Response of SRFA Compared to Peat-Derived DOM

SRFA, derived from the Okefenokee swamp and isolated via XAD-8 extraction, is characterized by CHO-dominated molecular formulas and lower aromatic carbon content relative to humic substances derived from solid organic deposits such as peat [4, 5]. In contrast, the peat-derived DOM samples analyzed here represent non-extracted material with higher structural heterogeneity and elevated contributions of heteroatom-containing formulas. Direct electrochemical reduction (DER) treatment of peat DOM resulted in increased proportions of hydrophobic formulas, consistent with redox-induced decreases in polarity [6], whereas SRFA, already enriched in hydrophobic fulvic components, exhibited limited compositional displacement. These compositional constraints likely contribute to the comparatively stable ionization efficiency of SRFA under ESI− conditions and the smaller TIC decrease observed between T₀ and T₁.

# Text S7: Calculation of Effective ΔDBE Equivalent to Measured EAC for the KR Sample

• EAC = 1132 μmol e⁻/gC

• ΔDBE_MS_= –0.62 (from MS analysis)

• Mean number of carbon atoms per molecule (waC) = 20.34 C atoms / molecule

M_C = 12 g mol^-1^ C

1) Electrons per molecule implied by ΔDBE_MS_

nₑ = –2 × ΔDBE_MS_ = –2 × (–0.62) = 1.24 e⁻ per molecule

(Each –1 in DBE corresponds to uptake of one H₂ equivalent = 2H⁺ + 2e⁻)

2) Effective ΔDBE equivalent to the measured EAC

n_e_ = EAC × waC × M_C × 10^-6^

Substituting values:

n_e_ = 1132 × 20.34 × 12 × 10^-6^ ≈ 0.276 mol e^-^ / mol molecule

Assuming two electrons per unit decrease in DBE:

ΔDBE_eff_ = -n_e_ / 2

ΔDBE_eff_ = – (1132 _×_ 20.34 × 12 × 10^-6^) / 2

ΔDBE_eff_ = -0.138

Thus, the EAC-equivalent change in DBE is: ΔDBEₑ_ff_ ≈ –0.138

Calculation of EAC and ΔDBEeff is based on the assumption of e^-^ transfer to C-atoms. Other elements e.g. O in quinones can also participate in redox reactions.

# Table S1: Description of sampling sites in this study.

| **Sample** | **Site Name** | **Location Description** | **Coordinates** | **Environment Type** | **Sampling Date** |
| --- | --- | --- | --- | --- | --- |
| SO | Sosa | A tributary of the Sosa drinking water reservoir (Ore Mountains of Saxony, Germany) [7]. | 50°28′17.7″ N,  12°40′14.3″ E | Mixed wetland/forest stream | August 2023 |
| GT | Goethemoor | Bog near the summit of Brocken Mountain, Harz Mountains, Germany [8]. | 51°47′23″ N,  10°36′6″ E | Mountain bog | 13.5.2024 |
| KR | Krycklan | Upstream of Catchment, ~50 km northwest of Umeå, northern Sweden [9]. | 64°25′0″ N,  19°46′30″ E | Fen | 26.9.2024 |

# Table S2: Intensity weighted-average (wa) carbon number and double bond equivalents (DBE), calculated for all molecular formulas in the KR-DOM sample, for untreated (T₀) and reduced (T₁) DOM across retention time (RT) segments and corresponding differences (Δ).

| **Retention time (min)** | **wa_C (T₀)** | **wa_C (T₁)** | **Δwa_C** | **wa_DBE (T₀)** | **wa_DBE (T₁)** | **Δwa_DBE** |
| --- | --- | --- | --- | --- | --- | --- |
| 9-10 | 18.0 | 17.8 | -0.2 | 9.2 | 8.3 | -0.9 |
| 10-11 | 18.4 | 18.1 | -0.3 | 10.0 | 9.0 | -1.0 |
| 11-12 | 19.5 | 19.1 | -0.4 | 10.8 | 9.7 | -1.1 |
| 12-13 | 20.0 | 19.6 | -0.4 | 11.2 | 10.0 | -1.2 |
| 13-14 | 20.3 | 19.9 | -0.4 | 11.5 | 10.5 | -1.0 |
| 14-15 | 20.5 | 20.1 | -0.4 | 11.8 | 10.8 | -1.0 |
| 15-16 | 21.0 | 20.7 | -0.3 | 11.4 | 10.4 | -1.0 |
| 16-17 | 21.2 | 20.9 | -0.3 | 11.2 | 10.1 | -1.1 |
| 17-18 | 21.5 | 21.2 | -0.3 | 10.6 | 9.7 | -0.9 |
| 18-19 | 22.0 | 21.6 | -0.4 | 9.9 | 8.8 | -1.1 |
| 19-20 | 22.4 | 22.0 | -0.4 | 9.1 | 8.0 | -1.1 |
| 20-21 | 23.5 | 23.0 | -0.5 | 7.9 | 7.0 | -0.9 |

# Table S3: Intensity-weighted average (wa) carbon number and double bond equivalents (DBE) for T_0_-unique, T_1_-unique, and common molecular formulas in the KR-DOM samples across selected retention-time windows (cf. Figure 5 in main text).

| **Retention time (min)** | **Formula class** | **wa_C** | **wa_DBE** |
| --- | --- | --- | --- |
| 10–11 | T_0_-unique | 20.45 | 11.75 |
| 10–11 | T_1_-unique | 16.57 | 8.35 |
| 10–11 | Common | 17.91 | 10.29 |
| 15–16 | T_0_-unique | 25.39 | 14.40 |
| 15–16 | T_1_-unique | 22.45 | 12.33 |
| 15–16 | Common | 21.74 | 10.98 |
| 20–21 | T_0_-unique | 23.20 | 10.07 |
| 20–21 | T_1_-unique | 19.50 | 7.53 |
| 20–21 | Common | 23.73 | 7.15 |

# Figure S1: Schematic of the two-compartment bulk electrolysis cell (50 mL) used for DER experiments. The working electrode (WE) and counter electrode (CE) compartments are separated by a frit and equipped with gas inlet/outlet ports. Adapted from Redoxme AB (Sweden).

# Figure S2: Chronoamperometric (i–t) response of AQDS (123 mg L⁻¹) and HNQ (216 mg L⁻¹). Raw current–time data are shown in grey, and smoothed traces are shown in orange (AQDS, −0.60 V vs Ag/AgCl) and green (HNQ, −0.39 V vs Ag/AgCl). Electron-accepting capacities (EACs) were determined by integration of the baseline-corrected i–t curves; dotted lines indicate the baseline.

# Figure S3: Representative chronoamperometric profiles (raw data) for GT, SO, and KR samples.

# Figure S4: *Comparison of electron accepting capacities (EAC) of DOM from this study with literature data. EAC values of DOM samples from this study were determined by direct electrochemical reduction and compared with mediated EAC values reported in the literature references [1, 2, 10, 11]*

# Figure S5: (a) Total ion chromatograms (TIC) profiles of the SO-DOM sample at two time points (T₀ in red, T₁ in blue). The curves, representing summed ion intensity as a function of chromatographic retention time, show a clear overall decrease in signal after DER. (b) Total ion chromatograms (TIC) profiles of the GT-DOM sample at two time points (T₀ in red, T₁ in blue). The summed ion intensity versus retention time likewise indicates a noticeable decrease in overall signal following electrochemical reduction.

# Figure S6: Total assigned chromatograms (TACs) of KR-DOM sample at two time points (T_0_, red; T_1_, blue), plotted as peak intensity versus chromatographic time.

# Figure S7: Total ion chromatograms (TICs) of Suwannee River Fulvic Acid (SRFA) at two time points: before reduction (T₀, red) and after reduction (T₁, blue).

# Figure S8: Intensity-weighted average double bond equivalent (DBE) versus number of carbon atoms derived for SO-DOM sample from LC-FT-ICR-MS data for T₀ (red circles) and T₁ (blue circles). Arrows indicate the direction of compositional change from T₀ to T₁. labels indicate the corresponding 1-min retention time from 10-11 to 21-22 min

# Figure S9: Intensity-weighted average H/C versus O/C for common formula between T_0_ and T_1_ derived from LC-FT-ICR MS data for KR-DOM sample. Segment numbers (1–12) correspond to 1-min retention-time intervals (e.g., 1 = 9–10 min, 2 = 10–11 min, … 12 = 20–21 min).

# Figure S10: Intensity-weighted average double bond equivalents (DBE) versus carbon number derived from LC-FT-ICR MS data for the KR sample. (a) Common molecular formulas detected in both T₀ (red) and T₁ (blue). Arrows indicate the direction of compositional change from T₀ to T₁ within each retention-time segment. (b) Unique molecular formulas detected in T₀ (red) or T₁ (blue). Segment numbers (1–12) correspond to 1-min retention-time intervals (e.g., 1 = 9–10 min, 2 = 10–11 min, … 12 = 20–21 min). Values represent intensity-weighted averages within each segment.

# Figure S11: Changes in DBE/C after DER for the KR-DOM sample. DBE/C values across LC retention time for T_0_ before DER and T_1_ after DER, calculated from (a) all detected formulas and (b) formulas common to both T_0_ and T_1_ within each retention time segment.

# Figure S12: (a) Log₂(T₁/T₀) intensity ratios (base peak–normalized) for molecular formulas detected in both untreated (T_0_) and electrochemically reduced (T_1_) KR-DOM, plotted against (a) H/C, (b) DBE-O, (c) O/C, and (d) AI_mod_. The dashed line indicates the TIC derived log_2_[(T_1_/T_0_) response, and dotted lines indicate the ±log_2_(1.5) threshold. Colors indicate formulas with a relative decrease compared with the TIC response (red), no clear change within the threshold range (gray), or relative increase (blue).

# References

1. Guth P, Gao C, Knorr KH. Electron accepting capacities of a wide variety of peat materials from around the globe similarly explain CO2 and CH4 formation. Global Biogeochem Cycles. 2023;37(1):e2022GB007459.

2. Walpen N, Getzinger GJ, Schroth MH, Sander M. Electron-donating phenolic and electron-accepting quinone moieties in peat dissolved organic matter: quantities and redox transformations in the context of peat biogeochemistry. Environ Sci Technol. 2018;52(9):5236-45.

3. Legendre P, Legendre L. Numerical ecology. Elsevier; 2012.

4. Hodgkins SB, Tfaily MM, Podgorski DC, McCalley CK, Saleska SR, Crill PM, et al. Elemental composition and optical properties reveal changes in dissolved organic matter along a permafrost thaw chronosequence in a subarctic peatland. Geochim Cosmochim Acta. 2016;187:123-40.

5. Herzsprung P, Hertkorn N, Von Tümpling W, Harir M, Friese K, Schmitt-Kopplin P. Molecular formula assignment for dissolved organic matter (DOM) using high-field FT-ICR-MS: chemical perspective and validation of sulphur-rich organic components (CHOS) in pit lake samples. Anal Bioanal Chem. 2016;408(10):2461-9.

6. Aeschbacher M, Vergari D, Schwarzenbach RP, Sander M. Electrochemical analysis of proton and electron transfer equilibria of the reducible moieties in humic acids. Environ Sci Technol. 2011;45(19):8385-94.

7. Charamba LV, Houska T, Kaiser K, Knorr K-H, Krüger S, Krause T, et al. Tracing sources of dissolved organic matter along the terrestrial-aquatic continuum in the Ore Mountains, Germany. Sci Total Environ. 2024;943:173807.

8. Czarnota P, Kison H-U, Seelemann A. Remarkable records of lichens and lichenicolous fungi from the Harz National Park (Lower Saxony and Saxony-Anhalt, Germany). Herzogia. 2014;27(1):67-82.

9. Laudon H, Taberman I, Ågren A, Futter M, Ottosson‐Löfvenius M, Bishop K. The Krycklan Catchment Study—A flagship infrastructure for hydrology, biogeochemistry, and climate research in the boreal landscape. Water Resour Res. 2013;49(10):7154-8.

10. Aeschbacher M, Sander M, Schwarzenbach RP. Novel electrochemical approach to assess the redox properties of humic substances. Environ Sci Technol. 2010;44(1):87-93.

11. Bauer M, Heitmann T, Macalady DL, Blodau C. Electron transfer capacities and reaction kinetics of peat dissolved organic matter. Environ Sci Technol. 2007;41(1):139-45.
